# Pre-Interventional Risk Assessment in The Elderly (PIRATE): Development of a scoring system to predict 30-day mortality using data of the Peri-Interventional Outcome Study in the Elderly

**DOI:** 10.1371/journal.pone.0294431

**Published:** 2023-12-21

**Authors:** Alina Schenk, Ana Kowark, Moritz Berger, Rolf Rossaint, Matthias Schmid, Mark Coburn, the POSE Study group

**Affiliations:** 1 Department of Medical Biometry, Informatics and Epidemiology, Faculty of Medicine, University of Bonn, Bonn, Germany; 2 Department of Anaesthesiology and Intensive Care Medicine, University Hospital Bonn, Bonn, Germany; 3 Department of Anaesthesiology, Medical Faculty University Hospital RWTH Aachen, Aachen, Germany; Universita degli Studi di Napoli Federico II, ITALY

## Abstract

Risk assessment before interventions in elderly patients becomes more and more vital due to an increasing number of elderly patients requiring surgery. Existing risk scores are often not tailored to marginalized groups such as patients aged 80 years or older. We aimed to develop an easy-to-use and readily applicable risk assessment tool that implements pre-interventional predictors of 30-day mortality in elderly patients (≥80 years) undergoing interventions under anesthesia. Using Cox regression analysis, we compared different sets of predictors by taking into account their ease of availability and by evaluating predictive accuracy. Coefficient estimates were utilized to set up a scoring system that was internally validated. Model building and evaluation were based on data from the Peri-Interventional Outcome Study in the Elderly (POSE), which was conducted as a European multicenter, observational prospective cohort study. Our risk assessment tool, named PIRATE, contains three predictors assessable at admission *(urgency*, *severity* and *living conditions*). Discriminatory power, as measured by the concordance index, was 0.75. The estimated prediction error, as measured by the Brier score, was 0.036 (covariate-free reference model: 0.043). PIRATE is an easy-to-use risk assessment tool that helps stratifying elderly patients undergoing interventions with anesthesia at increased risk of mortality. PIRATE is readily available and applies to a wide variety of settings. In particular, it covers patients needing elective or emergency surgery and undergoing in-hospital or day-case surgery. Also, it applies to all types of interventions, from minor to major. It may serve as a basis for multidisciplinary and informed shared decision-making.

## Introduction

According to the World Health Organization (WHO) World *Report on Aging and Health* significant impairment in the elderly population is reported. The number of elderly people in Europe will double by 2050 and thus the number of elderly patients requiring surgery [1]. In consequence, there is increasing need for pre-interventional risk assessment and outcome prediction focusing on elderly patients.

The key challenge of any pre-interventional risk assessment in the elderly is to identify and stratify patients at increased risk of mortality and morbidity, accounting for characteristics that are of particular importance to elderly people, like functional status, level of independence and frailty. Pre-interventional risk assessments may thus contribute to informed decision making, helping both, the patients and possible authorized representatives of the elderly patients, to better evaluate the trade-off between the medical necessity of a (non-)surgical intervention and patient specific outcomes [2]. Moreover, they may be employed to guide clinical planning and decision making, in particular by customising (non-)surgical interventions. In this respect, the updated *Pre-Operative Evaluation of Adults Undergoing Elective Noncardiac Surgery* guideline of the European Society of Anaesthesiology and Intensive Care recommends in its section on geriatric patients to assess *pre-*interventional functional status, level of independence, comorbidities and frailty [3]. Further, the guideline on *Perioperative Care in Adults* published by the National Institute for Health and Care Excellence (NICE) in 2020 recommends to use validated risk stratification tools to supplement clinical assessment when planning surgery [4].

Despite these recommendations, there are thus far no risk assessment tools specifically developed on elderly patients (≥80 years). To the best of our knowledge, no performance evaluations of existing risk assessment scores in the subgroup of elderly patients exist. Commonly used scores such as e.g. the *Preoperative Score to Predict Postoperative Mortality* (POSPOM), the *Physiological and Operative Severity Score for the Enumeration of Mortality and Morbidity* (POSSUM), the *Portsmouth-POSSUM* (P-POSSUM), the *Surgical Outcome Risk Tool* (SORT), the *National Surgical Quality Improvement Program* (NSQIP) *Universal Surgical Risk Calculator*, the *Estimation of Physiologic Ability and Surgical Stress* (E-Pass), and the *Surgical Risk Scale* (SRS) have all been developed on data referring to a wider age range and employing a number of risk factors that are, to some extent, not assessable before intervention [5–13].

Therefore, the aim of this analysis was to develop a pre-interventional risk calculation tool that is tailored to the assessment of post-interventional mortality in elderly patients (≥ 80 years). Using prospectively collected data from the Peri-interventional Outcome Study in the Elderly (POSE), we derived and internally validated a user-friendly scoring system, named **P**re-**I**nterventional **R**isk **A**ssessment in **T**he **E**lderly (PIRATE) [14]. As described in detail in the Results section below, PIRATE resulted from a stepwise predictor selection procedure taking into account

- simplicity and usability of the scoring system in daily clinical practice (avoiding complex and time-consuming calculations),
- ease of availability of predictors *before* intervention (in particular, by using unambiguously defined risk categories), and
- prediction accuracy.

Reporting of the PIRATE tool will be based on the *Transparent Reporting of a Multivariable Prediction Model for Individual Prognosis or Diagnosis* (TRIPOD) statement [15].

## Methods

### Study population

The step-by-step development of our scoring system was based on the POSE database (exported on 17^th^ of February 2020). POSE was conducted as a European multicenter, observational prospective cohort study to investigate mortality rates and other outcomes in the elderly population. Patients were eligible, if aged 80 years or older and undergoing surgical or non-surgical interventions under anesthesia. The study period lasted from October 2017 to December 2018. Each center recruited patients for 30 consecutive days within the study period. Interventions were classified as either surgical or non-surgical, elective or non-elective, and inpatient or outpatient. In total, POSE enrolled 9,862 patients from 177 study centers in 20 different countries, of which 9,497 patients were eligible for analysis. The reasons for exclusion of 365 patients comprised death before intervention (n = 20), intervention postponed/ cancelled (n = 301), missing patient records (n = 22), and not collected data (n = 22). Of 9,497 patients, 388 experienced the event of interest (i.e., death within 30 days after intervention) and 9,109 did not experience the event of interest (“controls”), resulting in a post-interventional mortality rate of 4.2% (95% CI 3.8%-4.7%) [14]. POSE was approved by the University Hospital RWTH Aachen, Germany (EK 162/17). Mandatory research ethics board (REB) approval or a waiver was granted at each center. Written informed consent was obtained from all subjects participating in the trial. POSE was registered prior to patient enrollment at clinicaltrials.gov (NCT03152734, Chief coordinating investigator: Mark Coburn, Date of registration: May 15, 2017). The development of PIRATE was approved by the POSE Steering Committee as a secondary analysis (https://pose-trial.org/secondary-analyses). A data transfer agreement between the University Hospital RWTH Aachen and the Department of Medical Biometry, Informatics and Epidemiology, Faculty of Medicine, University of Bonn was established. AS, MB and MS had no access to information that could identify individual patients during or after data collection. It is not precluded that AK, RR and MC could have identified patients from their respective study site in the course of their work as treating physicians.

### Outcome definition

The outcome of interest was the time after intervention until death from any cause. Patients potentially having an event after 30 days were censored. The survival status of patients discharged before day 30 was enquired using telephone interviews [14].

### Definition and choice of predictors

The aim of this secondary analysis was the pre-interventional risk assessment of post-interventional mortality of elderly patients (≥ 80 years), i.e., the prediction of 30-day mortality after intervention.

The basis of the stepwise development of the PIRATE tool was the complete POSE cohort (9,497 patients). We considered 15 potential predictors (seven binary, six categorical and two continuous predictors). Of these, ten predictors (four binary, four categorical and two continuous predictors) fulfilled the requirement of being assessable *before* intervention (see the POSE statistical analysis plan [14] for details on all available predictors and their categories, see Table 1 for details on included predictors and their categories). These ten predictors, including *age [years]*, *bmi [kg/m*^*2*^*]*, *sex*, *severity (minor*, *intermediate*, *major)* and *urgency (elective*, *urgent*, *emergent)* of intervention, *type of intervention*, *multimorbidity* and *referring facility* of the patients as well as *frailty* and a test for patients’ *mobility* (timed up and go [TUG] test) were considered in the development process of the scoring system. In POSE, a patient was classified as *frail* if at least 4 of 6 criteria (mini‐cog score of ≤ 3 points, albumin level of ≤ 3.3 g/d, more than 1 fall in the last 6 months, haematocrit level of < 35%, preoperative functional status is partially dependent or totally dependent, ≥3 comorbidities) were fulfilled [14]. Following the definition by the WHO, *multimorbidity* was defined as the presence of at least two chronic conditions [1, 14]. The TUG test was performed to assess mobility of patients. The patients were asked to stand up from a chair, to walk three metres, to turn around and to walk back and sit down again. The test result was evaluated as normal mobility if the patient was able to perform the TUG test in 12 seconds or less. If the patient was not able to perform the TUG test or took more than 12 seconds to perform the test, the test result was evaluated as limited mobility.

**Table 1:** Patient characteristics of the POSE [14] cohort used for the development of PIRATE. Values are mean (SD) or number (proportion).

Variable	All n = 9497 n (%)	Cases n = 388 n (%)	Controls n = 9109 n (%)
**Age [years]** (mean, sd)	84.32 (3.8)	85.78 (4.8)	84.26 (3.8)
**BMI [kg/m**^**2**^**]** (mean, sd)	25.94 (4.33)	25.15 (4.53)	25.98 (4.32)
Missing	148 (1.6%)	11 (2.8%)	137 (1.5%)
**Sex**			
male	4485 (47.2%)	192 (49.5%)	4293 (47.1%)
female	5012 (52.8%)	196 (50.5%)	4816 (52.9%)
**Severity**			
minor	1947 (20.5%)	38 (9.8%)	1909 (21.0%)
intermediate	3612 (38.0%)	107(27.6%)	3505 (38.5%)
major	3938 (41.5%)	243 (62.6%)	3695 (40.6%)
**Urgency**			
elective	7176 (75.6%)	146 (37.6%)	7030 (77.2%)
emergent	479 (5.0%)	87 (22.4%)	392 (4.3%)
urgent	1842 (19.4%	155 (39.9%)	1687 (18.5%)
**Frailty**			
frail	1336 (14.1%)	180 (46.4%)	1156 (12.7%)
not frail	8161 (85.9%)	208 (53.6%)	7953 (87.3%)
**Type of intervention**			
abdominal	1149 (12.1%)	89 (22.9%)	1060 (11.6%)
cardiovascular and thoracic	896 (9.4%)	60 (15.5%)	836 (9.2%)
ENT; ophthalmologic	1594 (16.8%)	8 (2.1%)	1586 (17.4%)
gynaecologic and urologic	1437 (15.1%)	21 (5.4%)	1416 (15.5%)
interventional	1026 (10.8%)	29 (7.5%)	997 (10.9%)
neurosurgery	196 (2.1%)	22 (5.7%)	174 (1.9%)
orthopaedic, trauma and plastic	2860 (30.1%)	142 (36.6%)	2718 (29.8%)
transplant or other surgery	339 (3.6%)	17 (4.4%)	322 (3.5%)
**Living conditions (Facility)**			
Home	8220 (86.6%)	254 (65.5%)	7966 (87.5%)
Other hospital	184 (1.9%)	31 (8.0%)	153 (1.7%)
Rehabilitation	60 (0.6%)	2 (0.5%)	58 (0.6%)
Nursing home	670 (7.1%)	65 (16.8%)	605 (6.6%)
other	360 (3.8%)	36 (9.3%)	324 (3.6%)
missing	3 (0.03%)	0 (0%)	3 (0.03%)
**Multimorbidity**			
yes	7334 (77.2%)	359 (92.5%)	6975 (76.6%)
no	2163 (22.8%)	29 (7.5%)	2134 (23.4%)
**Mobility (TUG test)**			
limited	6461 (68.0%)	316 (81.4%)	6145 (67.5%)
normal	1910 (20.1%)	16 (4.1%)	1894 (20.8%)
missing	1126 (11.9%)	56 (14.4%)	1070 (11.7%)

Abbreviation

BMI = Body Mass Index

ENT = Ear, Nose and Throat

POSE = Peri-Interventional Outcome Study in the Elderly

PIRATE = Pre-Interventional Risk Assessment in The Elderly

SD = Standard deviation

TUG = Timed up and go

### Development of the scoring system

Development of the scoring system was based on a stepwise procedure that accounted for the trade-off between prediction accuracy and simplicity, focussing on the predictors’ ease of availability in daily clinical routine. In each step of the development process, we fitted a Cox proportional hazards regression model containing different subsets or combinations of the ten initially available predictors (described above). In order to internally validate the developed scoring system at each step, we repeatedly divided the entire study cohort on the center level into a derivation cohort and a validation cohort (100 replications). Specifically, each derivation cohort provided a training data set comprising a set of randomly chosen study centers that included approximately two thirds of the patients in POSE. The patients of the remaining study centers were allocated to the respective validation cohort providing the test data set. Prediction accuracy was measured using the concordance index (C-index) averaged across the 100 validation cohorts [16]. Variable importance was measured by the loss in C-index when permuting the respective predictor. To assess calibration, we generated calibration plots that compared predicted 30-day survival probabilities to their respective Kaplan-Meier estimates. Prediction error of the final model was measured using the Brier score [17]. The various model building steps will be described in detail in the Results section. After model building, we developed a scoring system based on the final Cox proportional hazards regression model, assigning risk points to each category of the included risk factors (predictors) [18]. With this system (entitled **P**re-**I**nterventional **R**isk **A**ssessment in **T**he **E**lderly [PIRATE]), users can simply add all risk points and extract the respective estimated 30-day mortality from a look-up table.

### Handling of missing data

Missing data were imputed using multiple imputation (fully conditional specification with all ten initially available predictors [19, 20]). We generated 12 imputed data sets, following the POSE trial statistical analysis plan [14].

A sensitivity analysis composed of the application of the development process on each of the 12 imputed data sets revealed only marginal differences in the results (on the third decimal place of C-index values) that are less relevant for the final conclusions. Thus, the development is illustrated for one single imputed data set in the following. The majority of missing values was present in *mobility*, which is, as explained in the Results section, not considered in the final scoring system. Thus, changes across the imputed datasets for *mobility* were negligible.

All calculations were performed using the R language and environment for statistical computing (version 4.1.0).

## Results

Patient characteristics of the 9,497 POSE patients (without imputation of missing values) are presented in Table 1. In the following, we will give a detailed description of each model building step, weighing simplicity, usability, availability of predictors and prediction accuracy. The C-index value presented in each step represents the mean value averaged across 100 replications.

### Step 0: Model with all available predictors

The model including all initially available predictors (*age*, *bmi*, *sex*, *facility*, *type of intervention*, *severity*, *urgency*, *multimorbidity*, *timed up go*, *frailty*) reached a mean C-index of 0.818.

### Step 1: Grouping of predictors based on availability at the time of admission

Based on expert discussions with members of the POSE study team, we grouped the predictors according to the following criteria:

- Very easy to gather: *age*, *sex*, *facility*,
- Easy to gather: *bmi*, *urgency*, *type of intervention*,
- Hard to gather: *severity*, *multimorbidity*,
- Very hard to gather: *frailty (as assessed in POSE)*, *timed up go*.

Based on this grouping, we considered the following set of models:

- Model 0: *Null model (without any predictors)*,
- Model 1: *age*, *sex*, *facility*,
- Model 2: *age*, *sex*, *facility*, *bmi*, *urgency*, *type of intervention*,
- Model 3: *age*, *sex*, *facility*, *bmi*, *urgency*, *type of intervention*, *severity*, *multimorbidity*,
- Model 4 (from Step 0): *age*, *sex*, *facility*, *bmi*, *urgency*, *type of intervention*, *severity*, *multimorbidity*, *frailty*, *timed up go*.

**Fig 1:**
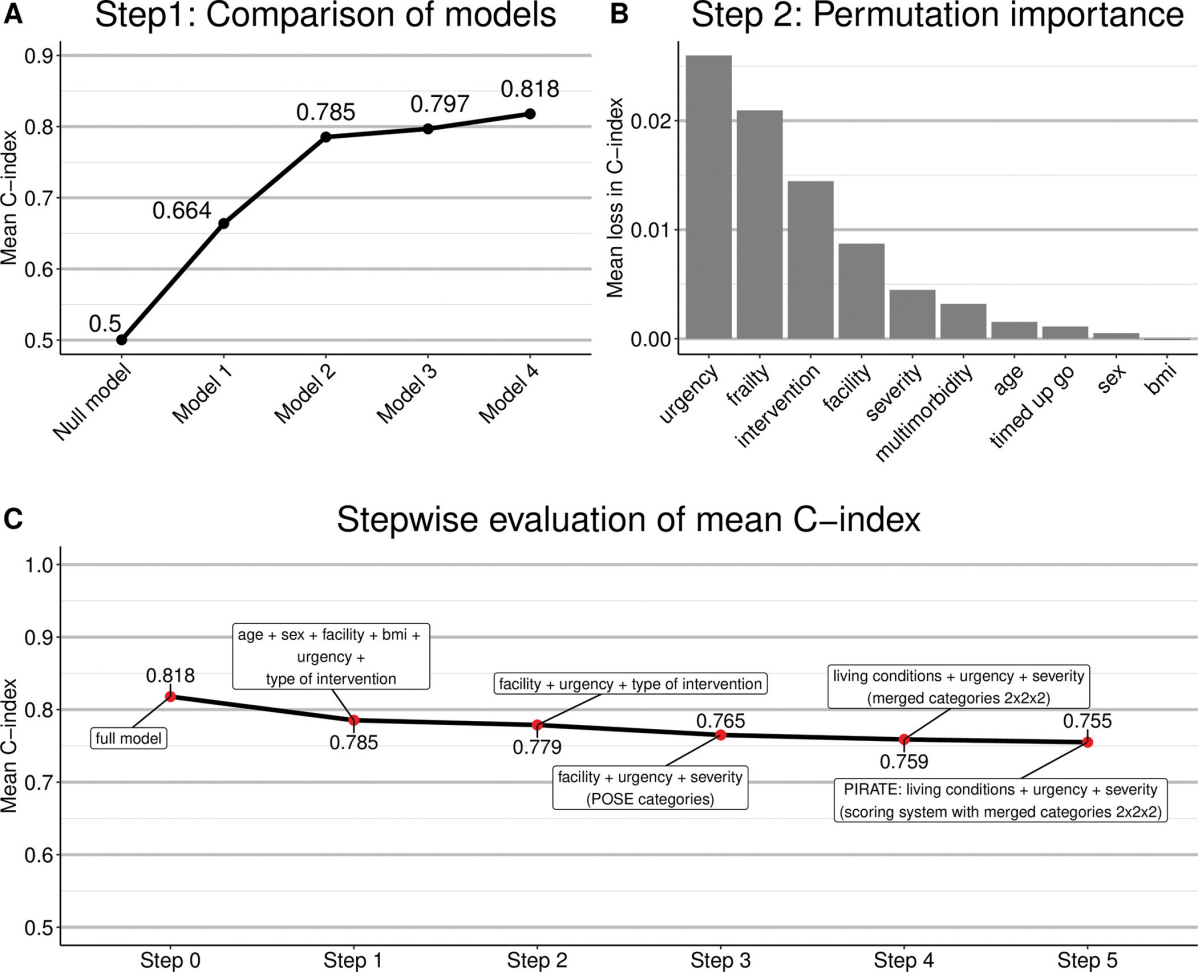
Development of PIRATE. (A) Mean C-index values that were obtained from applying the models in Step 1 to the 100 different training data sets. There was an upwards trend in prediction accuracy as the number of predictors increased in each model. (B) Permutation importance of the ten initially available predictors in Step 2. Permutation importance was defined as the difference between the C-index values obtained from the full model (from Step 0) with original data and the model(s) with permuted data. (C) Stepwise evaluation of the mean C-index from Step 0 (full model) to Step 5 (PIRATE).

Fig 1(A) presents the mean C-index values that were obtained from applying the above models to the 100 different training data sets. It is seen that there was an upwards trend in prediction accuracy as the number of predictors increased. On the other hand, the differences in C-index values between models 2, 3 and 4 were considerably smaller than the respective difference between models 1 and 2. Based on this result and keeping the ease of availability of the predictors in mind, model 2 (including *age*, *sex*, *facility*, *bmi*, *urgency* & *type of intervention*, *and excluding four predictors from Step 0)* seemed to be a reasonable compromise between prediction accuracy and usability. The mean C-index of model 2 was 0.785.

### Step 2: Statistical importance of the predictors (permutation importance)

In the next step, we analyzed the individual contributions of the ten predictors to the prediction accuracy of the models. To this purpose, we ranked the predictors according to their (statistical) permutation importance. This was done by randomly permuting the training data of the ten available predictors, considering one predictor at a time. Full models with all ten predictors were then fitted to the training data (one model per permuted predictor, each time leaving the training data of the other eight predictors unchanged) and the C-indices were calculated on the (non-permuted) test data. For each predictor, we calculated its permutation importance, which was defined as the difference between the C-index values obtained from the full model with original data and the model(s) with permuted data. The ranking of the importance values of the ten predictors is presented in Fig 1(B). Statistically, the most important predictor was *urgency* followed by *frailty*, *type of intervention* and *facility*. Including these four predictors in the model, we obtained a mean C-index of 0.807 (on 100 different test data sets containing one-third of the complete cohort). *Urgency* as well as *type of intervention* and *facility* matched the set of predictors contained in our favored model in Step 1. *Frailty*, however, was not considered for inclusion in this model, as it is rather hard to assess in clinical routine when using the definition of frailty in POSE (comprising six individual items, see [14]). Further, the inclusion of *age*, *bmi* and *sex* (and *timed up go*) did not result in a gain in the mean C-index compared to the model excluding those predictors. Additionally, the inclusion of *frailty* in Step 1 (Model 3 vs. Model 4) did not increase the C-index appreciably (0.797 compared to 0.818). Thus, excluding *frailty*, *age*, *bmi* and *sex*, we fitted a model solely containing *urgency*, *type of intervention* and *facility*. This model resulted in a mean C-index of 0.779 (compared to 0.785 for the model from Step 1 containing *age*, *sex*, *facility*, *bmi*, *urgency* and *type of intervention*; see Fig 1(C)).

### Step 3: Replacement of type of intervention by severity

The model from Step 2 containing *urgency*, *type of intervention* and *facility* consists of three categorical predictors with, in total, 3 x 8 x 5 = 120 combinations of categories. Regarding the simplicity and usability of the score in clinical routine, differentiating eight categories for *type of intervention* seems impractical given that the score should be calculated as quickly as possible. On the other hand, the *severity* of an intervention (coded by three categories) is strongly associated with the *type of intervention*: Once the *type of the intervention* is known, the *severity* of an intervention can simply be evaluated (Chi-Squared test, *p*<10^−16^). The replacement of *type of intervention* by *severity* in our model lead to a slightly lower mean C-index (0.765 compared to 0.779 from step 2 [including *urgency*, *type of intervention* and *facility*], Fig 1(C)) but tremendously facilitates the application of the score.

### Step 4: Merging categories

The model resulting from Step 3 containing *urgency*, *severity* and *facility* included three categorical predictors with 3 x 3 x 5 = 45 combinations of categories. In order to further simplify calculation of the score, we reduced the number of categories of each predictor to two. More specifically, we collapsed two of the three categories of *urgency* (*elective*, *urgent* and *emergency*), obtaining a binary predictor that indicated whether an intervention was planned (*elective*) or not. Analogously, rather than distinguishing between *minor*, *intermediate* and *major severity*, we generated a binary predictor indicating whether the intervention to be performed was *major* or not. *Referring facility* was transformed into the two categories *independently living* or *(medically) assisted*. Here, the categories *rehabilitation*, *other hospital* and *nursing home* were summarized to *(medically) assisted* while *home* was considered as *independently living*, since the respective field in the case report form was originally *home/independent*. Further, regarding the category *other* in *referring facility*, free text answers were manually screened and assigned to one of the two aforementioned categories. More specifically, free text answers (indicated as *other* in Table 1) referring to *religious community*, *monastery*, *hostel* and *homeless* were allocated to *independently living* while all other text answers indicated help from a family member or a trained nurse and were therefore allocated to *(medically) assisted*. In the remainder, we will use the term *living conditions* consisting of the two aforementioned aggregated categories instead of *facility* which refers to the covariate with the initial five categories as in POSE. The simplified score containing the three binary predictors reached a mean C-index of 0.759 (Fig 1(C)).

### Step 5: Transferring the score to a scoring system

To facilitate the application and interpretation of the score in the clinical practice, we transferred the model derived in Step 4 to a scoring system that is based on the assignment of *risk points*. Following the approach described in Sullivan et al. [18], we fitted Cox regression models to the data of the 100 derivation cohorts, incorporating the three binary predictors derived in Step 4. Based on the estimated coefficients obtained from the Cox regression models, the scoring system was set up in each of the derivation cohorts, and the respective estimated 30-day probabilities of death were calculated for the patients in the validation cohorts. Reference categories for each risk factor were chosen according to the strength of risk association, assigning zero points to the groups with the lowest risk and higher numbers of points to groups with higher risk (for details, see [18]). Thus, an increasing score is related to an increased estimated 30-day probability of death. We termed the resulting system **P**re-**I**nterventional **R**isk **A**ssessment in **T**he **E**lderly (PIRATE). Note that the methodology proposed by Sullivan et al. involves a constant *B* reflecting the number of regression units corresponding to one point [18]. For PIRATE, we set *B* equal to the regression coefficient of *severity*, as estimated from the Cox regression model. Thus, the constant reflects the increase in 30-day mortality risk associated with a major intervention [18].

Compared to the Cox regression model in Step 4, the C-index of the scoring system decreased only slightly (from 0.759 to 0.755, see Fig 1(C) and below).

### PIRATE: The final risk assessment tool

The final scoring system (complete cohort with 9,497 observations) is presented in Table 2. Using the data in Table 2, the individual risk of a patient can be calculated by summing up all points belonging to the values of the patient’s risk factors. The respective estimated 30-day probability of death can be extracted from the “look-up” Table 3. Total score values in the full POSE cohort ranged between 0 and 5 (see S1 Table for example calculations of the risk score).

**Table 2:** PIRATE scoring system, as derived from the coefficient estimates of the Cox regression model in Step 4. The constant B is given as *B* = 0.5986 [18].

Risk factor	Coefficient estimate	Risk points
**Severity**		
minor/intermediate	-	+0
major	0.5986	+1
**Urgency**		
elective	-	+0
non-elective	1.3912	+2
**Living conditions**		
independent	-	+0
(medically) assisted	0.8985	+2

Abbreviation

PIRATE = Pre-Interventional Risk Assessment in The Elderly

**Table 3:** Look-up table for the predicted 30-day probability of death after intervention.

Total points	Estimated probability of death [%] (within 30 days after intervention)
0	1.29%
1	2.33%
2	4.20%
3	7.51%
4	13.24%
5	22.78%

**Fig 2:**
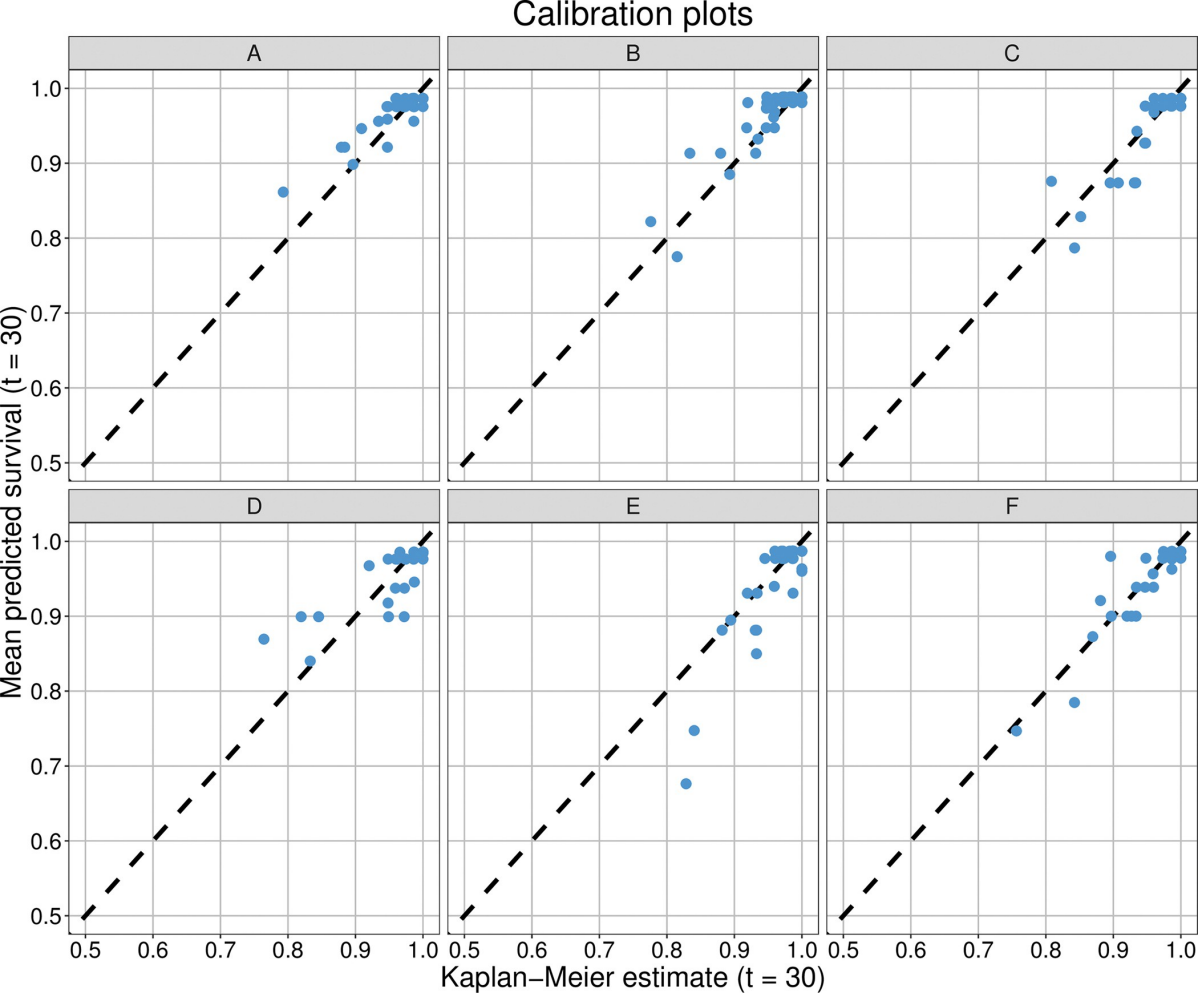
Calibration plots. Calibration plots for six exemplary validation cohorts. The plots depict the predicted probabilities based on the scoring system versus the Kaplan-Meier estimates in subgroups.

The scoring system showed good discrimination ability with the mean estimated C-index across all validation cohorts of 0.755 (min = 0.708, max = 0.797). Prediction error was also small, with mean estimated Brier score of 0.036 (min = 0.026, max = 0.046) across all validation cohorts (compared to 0.043 obtained from a reference model not containing any predictor information). Fig 2 shows exemplary calibration plots for six validation cohorts.

**Fig 3:**
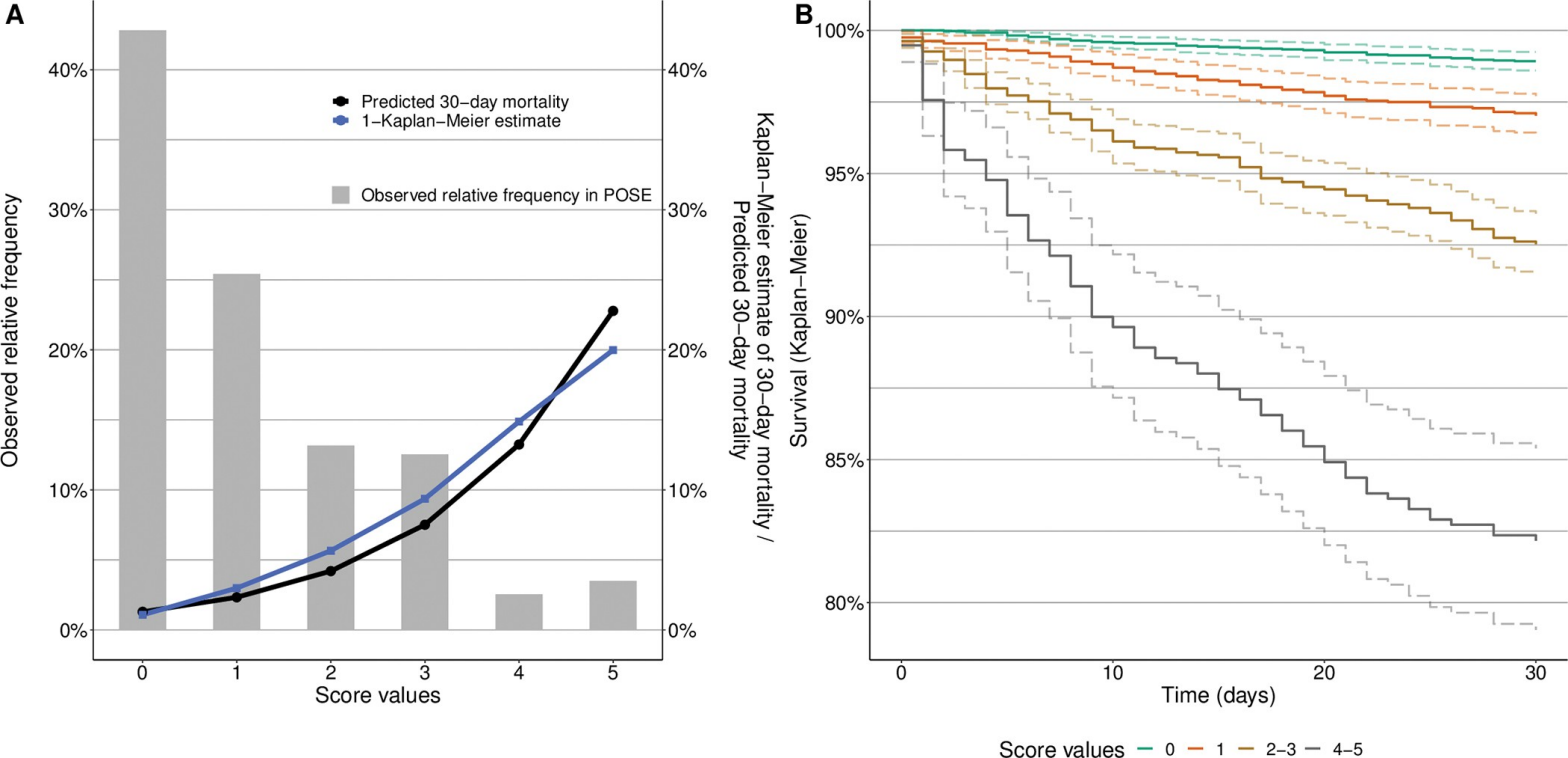
Evaluation of the PIRATE tool. (A) Distribution of the risk score values. The grey bars represent the relative frequencies of the risk score values in the full POSE cohort, the black line represents the respective estimated probabilities obtained from PIRATE, and the blue line refers to the death probabilities (one minus Kaplan-Meier estimates) for patients having the respective score. (B) Stratified Kaplan-Meier estimates in subgroups. Groups were defined by the 25%, 50% and 75% percentiles of the risk score values in POSE. The non-overlapping survival curves reflect the score’s ability to distinguish among high risk and low risk patients.

Fig 3(A) presents the distribution of the score values in the complete cohort (9,497 observations). The grey bars represent the relative frequencies of the score values in the full POSE cohort, the black line represents the respective estimated 30-day probabilities of death, and the blue line refers to the Kaplan-Meier estimates of 30-day mortality in patients having the respective score value. As seen from the figure, the scores in the POSE cohort mainly ranged between 0 and 3, with only few observations having a score higher than 3. Fig 3(A) shows that PIRATE-based probability estimates (black line) and the Kaplan-Meier estimates of 30-day mortality (blue line) matched well for almost all score values in the full POSE cohort.

Stratified Kaplan-Meier estimates in subgroups defined by the 25%, 50% and 75% percentiles of the score values are shown in Fig 3(B). Together with Fig 3(A), the non-overlapping survival curves in Fig 3(B) reflect the score’s ability to discriminate between high-risk and low-risk patients.

## Discussion

Using the POSE cohort, we were able to derive a new mortality risk assessment tool (PIRATE) that is based on three fast and simply to gather pre-interventional predictors. Starting with a multivariable Cox model containing ten predictors, our modeling approach balanced between (i) simplicity and usability of the score in clinical routine, (ii) availability of predictors (and the speed in gathering those) and (iii) prognostic accuracy of the score. The resulting PIRATE system demonstrates that an easy-to-use score solely based on readily available pre-interventional patient characteristics can be a powerful tool in predicting the post-interventional 30-day probability of death in elderly patients. In our internal validation analysis of the POSE data set, the three-predictor PIRATE system was able to identify patients with an increased mortality risk and discriminated well between high- and low-risk patients, thereby offering the possibility to improve both risk communication (based on easily understandable patient characteristics) and post-interventional treatment optimization. In particular, PIRATE highlights the markedly different prognoses for urgent (non-elective) and scheduled (elective) interventions. This is seen, for example, by considering the group of patients living medically assisted and undergoing a severe intervention (patients 3 and 4 in S1 Table): In this group, the predicted 30-day mortality risk is almost three times higher (22.78%) if the intervention is non-elective (patient 4) than if the intervention is elective (30-day mortality risk 7.51%, patient 3).

### Comparison to existing scores

Previously developed scores (e.g. POSSUM, P-POSSUM, POSPOM) used a logistic regression model with a binary outcome (dead vs. alive) for score development not accounting for censoring. In contrast to these scores, PIRATE is based on a Cox regression model that accounts for the characteristics of the survival and censoring processes during the post-interventional 30-day period [5–7]. Further, compared to other scores, we solely included readily available *pre*-interventional predictors, focussing on a quick and easy risk assessment before intervention [6, 7]. Similar to POSPOM, we derived a user-friendly scoring system that is applicable in daily clinical routine [5]. Of note, PIRATE was derived using data exclusively collected in the elderly target population.

As part of our project, we evaluated the predictive performance of the POSPOM scoring system in the POSE study cohort, mapping the categories in POSE to the risk factors used in POSPOM [5]. While POSPOM showed excellent performance and calibration on its original validation cohort extracted from the French National Hospital Discharge Database (C-index: 0.929), it reached a C-index of 0.76 in our study population containing elderly patients, which is, in fact, very similar to the C-index obtained from our PIRATE system (C-index: 0.755). In this respect, it is important to note that POSPOM was not developed exclusively for elderly patients, using a derivation cohort with mean age 54.6 years (SD = 17.9 years) and a slightly different outcome definition (all-cause mortality, regardless of whether in-hospital or not) [5].

Thus, our results demonstrate that, by optimising our system on data containing elderly patients only, and focussing on three simple pre-interventional factors, we were able to obtain essentially the same discriminatory power as the more complex POSPOM system.

### Prognostic predictors not included in PIRATE

The recently published updated guideline from the European Society of Anaesthesiology and Intensive Care Medicine recommends to assess pre-interventional functional status, level of independence, comorbidity and frailty in the geriatric patient [3]. The PIRATE easy-to-use characteristic *living conditions* is in line with this guideline. While developing PIRATE, we additionally analyzed several pre-interventional patient specific characteristics recommended in the guideline such as *frailty*, and the *type of the planned intervention* whose inclusion in a scoring system might lead to an even more accurate prediction of the post-interventional 30-day probability of death in elderly patients. Although increasing the prognostic power, which is in line with the recommendations of the guideline, those characteristics were not considered for PIRATE for different reasons as outlined in the Results section (i.e. ease of pre-interventional availability and the speed in gathering those) but have been described in previous risk prediction tools [5–13]. Regarding the assessment of frailty, it should be noted that several novel tools with a high accuracy and feasibility have become available during the past years [21]. These include, among others, the clinical frailty scale (CFS) [22], which has been systematically reviewed and recommended for use when predicting mortality and non-home discharge after surgery [23]. Since the CFS and its properties had not been studied in detail at the time POSE was planned, and since it was not possible to gather the CFS data retrospectively, we considered the original POSE frailty score for potential inclusion in PIRATE. The relatively large number of variables needed for the calculation of this score (both clinical and laboratory, see Methods section) led us to the decision to classify frailty as *very hard to gather*. In future studies involving the CFS, frailty will likely be much easier to assess.

Comprehensive geriatric assessment of elderly patients is generally considered to be important for the prognosis of post-interventional 30-day mortality. This has been demonstrated, for instance, by Abete et al. [24], who investigated the impact of surgical scores (e.g., POSSUM), living conditions, disabilities, cognitive function (evaluated by Mini-Mental State Examination, MMSE), depressive symptoms and the severity of comorbidities on 30-day mortality. In line with our results, they demonstrated that POSSUM (developed for patients undergoing emergency and elective surgical procedures, similar to PIRATE) and living conditions (included in the final PIRATE tool) were significantly associated with the 30-day mortality in patients aged 65 years or older [24]. While POSE also collected information on cognitive function (e.g. via the mini-cog test), we did not include these predictor variables in PIRATE, as we aimed to consider only those predictors that are readily available in emergency settings (see above). In this respect, it should be noted that the study setting considered by Abete et al. differed from POSE not only by the wider age range but also by the exclusion of patients with indication for emergency surgery. The evaluation procedures recommended by Abete et al. could thus be used as a tool to refine PIRATE in non-emergency cases.

Another important risk factor for post-interventional death is sarcopenia [25]. As sarcopenia is characterized by age-related loss of muscle mass and strength, it has been suggested to collect information on falls in elderly study populations and investigate the association between muscle mass, strength, and the prevalence of falls. In a comprehensive evaluation of non-institutionalized people, Curcio et al. [25] demonstrated a strong relationship between the Tinetti Mobility Test (TMT, being an indicator of fall risk) and muscle mass and strength, concluding that TMT represents a tool to detect sarcopenia in elderly patients [25]. In POSE, the mobility of elderly patients was evaluated by the history of falls, and also by the TUG test (both used in the *frailty* assessment). While we considered *frailty* in the development process of PIRATE, we eventually excluded this variable from the set of predictors, as it would be hard to gather the respective information in non-elective interventional settings (please see Step 2, and also the above discussion).

### Strengths

The development of the PIRATE scoring system is based on POSE, which was a prospective European multicenter study involving 177 hospitals across 20 countries. As a consequence, PIRATE refers to a broad study population while, at the same time, benefiting from quality-controlled data at the individual patient level collected in a highly standardized setting. We believe that this setting greatly improved estimation and prediction accuracy of the developed scores, even in view of a relatively moderate sample size (at least compared to often-used electronic health record databases involving more patients but employing less standardized methods for data capture).

Generally, the Cox regression model used in the development of PIRATE involves meaningful regression coefficients that have an intuitive interpretation in terms of hazard ratios, relating estimates to established formulas for the derivation of death probabilities. In particular, the use of Cox regression enabled us to translate the estimated regression coefficients into the proposed scoring system [18]. We acknowledge that the prediction accuracy of PIRATE might be improved further by replacing Cox regression with a machine-learning-(ML)-based technique. For example, recent work by Kwon et al. [26] and Seki et al. [27] indicated a strong performance of deep neural networks, random forests, multilayer perceptron and gradient boosting decision trees when used for the prediction of (in-hospital) mortality. However, while increasing prediction accuracy, ML-based predictions often rely on a multitude of predictor variables, which might–or might not–be assessable at the time of surgery. Also, they typically result in “black-box predictions”, complicating the interpretation of the predictors’ effects and requiring additional electronic support to make predictions on unseen data (e.g., through an online calculator). In contrast, PIRATE has the advantage of being readily applicable without having to use supplementary electronic tools.

By construction of the scoring system, PIRATE allows clinicians to assign risk points to the values of predictors at the individual patient level, including an immediate interpretation of which predictor indicates a worse outcome (e.g. a non-elective surgery leads to a higher probability of post-interventional death within 30 days than an elective one). Basing risk assessment on the scoring system instead of directly computing probabilities of death from the underlying Cox regression model may thus help to improve clinical utility and to establish the tool in daily clinical routine.

Common issues in score development are the transferability to and the external validation on different cohorts. These issues may become a problem when there are non-overlapping sets of risk factors in the derivation and validation cohorts, caused e.g. by different definitions or categorizations of predictors in the respective databases. These problems clearly do not apply to PIRATE, which guarantees a high degree of transferability due to its small number of unambiguously defined and easy-to-determine predictors.

### Limitations

Although the PIRATE tool has a number of distinct strengths, there are several limitations to consider. Compared to the development of POSPOM, for instance, which was based on data of 2,717,902 patients with 12,786 in hospital deaths (derivation cohort), the sample size and especially the number of events in the POSE cohort is relatively small [5]. On the other hand, as mentioned earlier, POSE provides prospectively collected data as part of a multicenter study ensuring high data quality compared to routinely collected data.

Importantly, we highlight the need for an external validation of the proposed scoring system. Although we performed an in-depth internal assessment of discrimination and calibration by repeatedly dividing the original POSE cohort on center level into a derivation and validation cohort, we acknowledge that selecting a prediction model based on comparisons of a performance measure (such as the C-index) is not guaranteed to be entirely free of some remaining “optimistic bias”. In this respect, external validation studies involving future or unseen data will provide further important insight in the generalization properties of PIRATE. We expect the collaborative network established for the POSE study (involving more than 170 study sites all over Europe) to facilitate the planning and conduct of such studies.

## Conclusions

In summary, the proposed PIRATE system constitutes a user-friendly tool to identify patients aged 80 years and older at increased risk of mortality after surgical intervention under anesthesia. PIRATE is readily available and applies to a wide variety of settings. In particular, it covers patients in need for elective or emergency surgery and undergoing in-hospital or day-case surgery. Also, it applies to all types of interventions, from minor to major. Further, PIRATE is in line with recent guidelines, which recommend to apply risk stratification tools to guide anesthesia care in the elderly patient. The scoring system could be used by physicians to evaluate patients‘ individual risk in order to adapt and customize treatment strategies and post-interventional health care. Future research needs to include an external validation of the scoring system.

## Supporting information

S1 File POSE study group. (DOCX)

S1 Table Example application of the PIRATE tool. (DOCX)

## References

[1] World Health Organization. World report on ageing and health. World Health Organization. 2015

[2] Oresanya LB, Lyons WL, Finlayson E. Preoperative assessment of the older patient: a narrative review. JAMA. 2014;311(20):2110–2120. doi: 10.1001/jama.2014.4573 24867014

[3] De Hert S, Staender S, Fritsch G, Hinkelbein J, Afshari A, Bettelli G, et al. Pre-operative evaluation of adults undergoing elective noncardiac surgery: Updated guideline from the European Society of Anaesthesiology. Eur J Anaesthesiol. 2018;35(6):407–465. doi: 10.1097/EJA.0000000000000817 29708905

[4] Perioperative care in adults. London: National Institute for Health and Care Excellence (NICE); August 19, 2020. 32931177

[5] Le Manach Y, Collins G, Rodseth R, Le Bihan-Benjamin C, Biccard B, Riou B, et al. Preoperative Score to Predict Postoperative Mortality (POSPOM): Derivation and Validation. Anesthesiology. 2016;124(3):570–579. doi: 10.1097/ALN.0000000000000972 26655494

[6] Copeland GP, Jones D, Walters M. POSSUM: a scoring system for surgical audit. Br J Surg. 1991;78(3):355–360. doi: 10.1002/bjs.1800780327 2021856

[7] Tyagi A, Nagpal N, Sidhu DS, Singh A, Tyagi A. Portsmouth physiological and operative severity score for the Enumeration of Mortality and morbidity scoring system in general surgical practice and identifying risk factors for poor outcome. J Nat Sci Biol Med. 2017;8(1):22–25. doi: 10.4103/0976-9668.198342 28250670 PMC5320818

[8] Wong DJN, Oliver CM, Moonesinghe SR. Predicting postoperative morbidity in adult elective surgical patients using the Surgical Outcome Risk Tool (SORT). Br J Anaesth. 2017;119(1):95–105. doi: 10.1093/bja/aex117 28974065

[9] Golan S, Adamsky MA, Johnson SC, Barashi NS, Smith ZL, Rodriguez MV, et al. National Surgical Quality Improvement Program surgical risk calculator poorly predicts complications in patients undergoing radical cystectomy with urinary diversion. Urol Oncol. 2018;36(2):77.e1–77.e7. doi: 10.1016/j.urolonc.2017.09.015 29033195

[10] Haga Y, Ikei S, Ogawa M. Estimation of Physiologic Ability and Surgical Stress (E-PASS) as a new prediction scoring system for postoperative morbidity and mortality following elective gastrointestinal surgery. Surg Today. 1999;29(3):219–225. doi: 10.1007/BF02483010 10192731

[11] Sutton R, Bann S, Brooks M, Sarin S. The Surgical Risk Scale as an improved tool for risk-adjusted analysis in comparative surgical audit. Br J Surg. 2002;89(6):763–768. doi: 10.1046/j.1365-2168.2002.02080.x 12027988

[12] Capuano AW, Shah RC, Blanche P, Wilson RS, Barnes LL, Bennett DA, et al. Derivation and validation of the Rapid Assessment of Dementia Risk (RADaR) for older adults. PLoS One. 2022;17(3):e0265379. doi: 10.1371/journal.pone.0265379 35299231 PMC8929636

[13] Piccininni M, Rohmann JL, Huscher D, Mielke N, Ebert N, Logroscino G, et al. Correction: Performance of risk prediction scores for cardiovascular mortality in older persons: External validation of the SCORE OP and appraisal. PLoS One. 2020;15(5):e0233051. doi: 10.1371/journal.pone.0233051 32374778 PMC7202620

[14] POSE-Study group. Peri-interventional outcome study in the elderly in Europe: A 30-day prospective cohort study. Eur J Anaesthesiol. 2022;39(3):198–209. doi: 10.1097/EJA.0000000000001639 34799496 PMC8815832

[15] Moons KG, Altman DG, Reitsma JB, Ioannidis JPA, Macaskill P, Steyerberg EW, et al. Transparent Reporting of a multivariable prediction model for Individual Prognosis or Diagnosis (TRIPOD): explanation and elaboration. Ann Intern Med. 2015;162(1):W1–W73. doi: 10.7326/M14-0698 25560730

[16] Gerds TA, Kattan MW, Schumacher M, Yu C. Estimating a time-dependent concordance index for survival prediction models with covariate dependent censoring. Stat Med. 2013;32(13):2173–2184. doi: 10.1002/sim.5681 23172755

[17] Kvamme H., & Borgan Ø. The Brier Score under Administrative Censoring: Problems and a Solution. J Mach Learn Res. 2023;24(2):1–26.

[18] Sullivan LM, Massaro JM, D’Agostino RB Sr. Presentation of multivariate data for clinical use: The Framingham Study risk score functions. Stat Med. 2004;23(10):1631–1660. doi: 10.1002/sim.1742 15122742

[19] Rubin DB. Multiple Imputation for nonresponse in surveys. New York, NY: Wiley; 1987.

[20] van Buuren S, Groothuis-Oudshoorn K. mice: Multivariate Imputation by Chained Equations in R. Journal of Statistical Software, 2011;45(3), 1–67.

[21] Alkadri J, Hage D, Nickerson LH, Scott LR, Shaw JF, Aucoin SD, et al. A Systematic Review and Meta-Analysis of Preoperative Frailty Instruments Derived From Electronic Health Data. Anesth Analg. 2021;133(5):1094–1106. doi: 10.1213/ANE.0000000000005595 33999880

[22] Guidet B, de Lange DW, Boumendil A, Leaver S, Watson X, Boulanger C, et al. The contribution of frailty, cognition, activity of daily life and comorbidities on outcome in acutely admitted patients over 80 years in European ICUs: the VIP2 study. Intensive Care Med. 2020;46(1):57–69. 31784798 10.1007/s00134-019-05853-1 PMC7223711

[23] Aucoin SD, Hao M, Sohi R, Shaw J, Bentov I, Walker D, et al. Accuracy and Feasibility of Clinically Applied Frailty Instruments before Surgery: A Systematic Review and Meta-analysis. Anesthesiology. 2020;133(1):78–95. doi: 10.1097/ALN.0000000000003257 32243326

[24] Abete P, Cherubini A, Di Bari M, Vigorito C, Viviani G, Marchionni N, et al. Does comprehensive geriatric assessment improve the estimate of surgical risk in elderly patients? An Italian multicenter observational study. Am J Surg. 2016;211(1):76–83.e2. doi: 10.1016/j.amjsurg.2015.04.016 26116322

[25] Curcio F, Basile C, Liguori I, Della-Morte D, Gargiulo G, Galizia G, et al. Tinetti mobility test is related to muscle mass and strength in non-institutionalized elderly people. Age (Dordr). 2016;38(5–6):525–533. doi: 10.1007/s11357-016-9935-9 27566307 PMC5266213

[26] Kwon JM, Kim KH, Jeon KH, Lee SE, Lee HY, Cho HJ, et al. Artificial intelligence algorithm for predicting mortality of patients with acute heart failure. PLoS One. 2019;14(7):e0219302. doi: 10.1371/journal.pone.0219302 31283783 PMC6613702

[27] Seki T, Kawazoe Y, Ohe K. Machine learning-based prediction of in-hospital mortality using admission laboratory data: A retrospective, single-site study using electronic health record data. PLoS One. 2021;16(2):e0246640. doi: 10.1371/journal.pone.0246640 33544775 PMC7864463

