# Supplementary material for: Pre-Interventional Risk Assessment in The Elderly (PIRATE): Development of a scoring system to predict 30-day mortality using data of the Peri-Interventional Outcome Study in the Elderly
Source: PLoS One. 2023 Dec 21;18(12):e0294431. doi: 10.1371/journal.pone.0294431 (PMC10734910; doi:10.1371/journal.pone.0294431)
Supplement: S1 File — (DOCX) [file pone.0294431.s001.docx]

**Supplemental Digital Content**

**S1. POSE-Study group.**

**POSE Study Management**

Chief Coordinating Investigator and Study Director:

Prof. Dr. med. Mark Coburn; Department of Anaesthesia and Intensive Care Medicine

University Hospital Bonn, Venusberg-Campus 1, 53127 Bonn, Germany

Investigator`s Representative and Study Coordinator:

Ana Kowark, Department of Anaesthesiology, Medical Faculty University Hospital RWTH Aachen, Aachen, Germany and Department of Anaesthesia and Intensive Care Medicine

University Hospital Bonn, Bonn, Germany

**Biostatistician of the main POSE-Study**

Prof. Dr. rer. nat. Ralf-Dieter Hilgers

Dr. Marcia V. Rückbeil

Department of Medical Statistics, Medical Faculty University Hospital RWTH Aachen, Aachen, Germany

**POSE Steering Committee**

Federico Bilotta, Department of Anaesthesiology and Intensive Care, University of Rome "La Sapienza", Rome, Italy

Cornelius Bollheimer, Department of Geriatric Medicine, Medical Faculty University Hospital RWTH Aachen, Aachen, Germany

Wolfgang Buhre, Department of Anaesthesiology and Pain Medicine, Maastricht University Medical Center, Maastricht, Netherlands

Ulf Günther, Department of Anaesthesiology and Intensive Care, University Hospital Oldenburg, Oldenburg, Germany

Andreas Hoeft, Department of Anaesthesiology and Intensive Care, University Hospital Bonn, Bonn, Germany (author deceased after finalisation of the manuscript)

Peter Lee, Department of Anesthesiology, Bon Secours Hospital, Cork, Ireland

Idit Matot, Anaesthesia, Pain and Intensive Care Division, Tel Aviv Sourasky Medical Center, Tel Aviv, Israel

Steffen Rex, Department of Anaesthesiology, UZ Leuven and Department of Cardiovascular Sciences, KU Leuven, Leuven, Belgium

Rolf Rossaint, Department of Anaesthesiology, Medical Faculty University Hospital RWTH Aachen, Aachen, Germany

Jacob Steinmetz, Department of Anaesthesiology, HOC 4231, Rigshospitalet, Copenhagen, Denmark

Jos Tournoy, Division of Gerontology and Geriatrics, University Hospital Leuven, Belgium and Department of Public Health and Primary Care, Leuven, Belgium

**POSE Collaborators (POSE Collaborators of the participating centres sorted by alphabetical order of country and city)**

**Austria**

*National Coordinator:*

Marc Berger, Department of Anaesthesiology, University Hospital Salzburg, Salzburg and Department of Anaesthesiology, University Hospital Essen, Essen, Germany

Department of Anaesthesiology, University Hospital Salzburg, Salzburg:

Marc Berger*, Helmut Farcher, Mathias Opperer; (*further affiliation: Department of Anaesthesiology, University Hospital Essen, Essen, Germany)

**Belgium**

*National Coordinator:*

Steffen Rex, Department of Anaesthesiology, University Hospital Leuven, Leuven

Department of Anaesthesiology, University Hospital Antwerp, Antwerp:

Ine Adriaensens, Vera Saldien

Department of Anaesthesiology, ZNA Middelheim, Antwerp:

Johan Berghmans, Sofie Van Hove

Department of Anaesthesiology, Ziekenhuis Oost-Limburg, Genk:

Maud Beran, Gert-Jan Eerdekens, Dieter Mesotten, Maxim Timmers, Elly Vandermeulen

Department of Anaesthesiology, Gent University Hospital, Gent:

Ann De Bruyne, Stefan De Hert, Hendrik De Ruyter

Department of Anaesthesiology, AZ Groeninge, Kortrijk:

Vincent Van Belleghem, Isabelle Boscart, Wouter De Corte, Matthias Desmet, Carlo Missant, Stefaan Carlier, Charlotte Castelain, Caroline Demeyer, Carl Vandenbossche

Department of Cardiovascular Sciences, Kulak Kortrijk Campus, Kortrijk:

Carlo Missant

Department of Anaesthesiology, University Hospital Leuven, Leuven:

Hans Detienne, Sarah Devroe, Geertrui Dewinter, Danny Hoogma, Christel Huygens, Roselien Meeusen, Steffen Rex, Marc Van de Velde

Department of Anaesthesiology, AZ Turnhout, Turnhout:

Christophe Lebrun, Stéphanie Poels, Filiep Soetens

**Denmark**

*National Coordinator:*

Lars H. Lundstrøm, Department of Anaesthesiology and Intensive Care, Nordsjællands Hospital, Hillerød

Department of Anaesthesiology, Aarhus University Hospital, Aarhus:

Christian Fenger-Eriksen

Department of Orthopaedics, Rigshospitalet, Copenhagen:

Christina Draegert, Sofia Gaspar Santos, Christine Soelling, Jacob Steinmetz

Department of Anaesthesiology, Herlev Hospital, University of Copenhagen, Herlev:

Gertrud Andersen, Sille Mølvig Dalsø, Pernille Haderslev, Vibe Maria Rasmussen, Morten Vester-Andersen

Department of Anaesthesiology and Intensive Care, Nordsjællands Hospital, Hillerød:

Tine Gjedde Sommer, Johan Kirkegaard, Lars H. Lundstrøm

Department of Anaesthesiology, Perioperative and Pain management, Hospital Unit Horsens, Horsens:

Christian Melchior Olesen, Sansu Paramanathan, Lisbet Tokkesdal Jensen

Department of Anaesthesiology, Vejle Hospital, Sygehus Lillebælt, Vejle:

Halfdan Holger Knudsen, Jens Christian Schmidt, Nick Pfaff Steen

**France**

*National Coordinator:*

Serge Molliex, Department of Anaesthesiology and Critical Care Medicine, University Hospital Saint-Etienne Nord, Saint Etienne

Department of Anaesthesiology, University Hospital Amiens Picardie, Amiens:

Hervé Dupont, Clément Herbinet, Emmanuel Lorne, Yazine Mahjoub, Alexandre Ntouba

Department of Anaesthesiology, University Hospital Angers, Angers:

Marine Fritsch, Manuela Garcia, Sigismond Lasocki, Jonathan Petit Phan

Department of Anaesthesiology, Center Hospitalier Fleyriat, Bourg en Bresse:

Thomas Lieutaud

Department of Anaesthesiology, Pierre Oudot Hospital Center, Bourgoin-Jallieu:

Laura Bonneric, Emmanuel Boselli, Maxime Gaillet

Department of Anaesthesiology, Military Hospital Clermont Tonnerre, Brest:

Marc Danguy des Déserts, Etienne Montelescaut

Department of Anaesthesiology and Intensive Care, Military Hospital Percy, Clamart:

Antoine Lamblin, Violaine Muller

Department of Anaesthesiology, Hospital Center Le Mans, Le Mans:

Celine Lagrange, Charlene Le Moal, Alain Robert, Frederik Staikowsky

Department of Anaesthesiology and Intensive Care, Hôpital Huriez of University Hospital Lille, Lille:

Benoit Lebas, Gilles Lebuffe, Matthias Garot

Department of Anaesthesiology and Intensive Care, Clinique de la Sauvegarde, Lyon:

Johanne Beuvelot, David Dejour, Emmanuel Deligne, Olivier Desebbe, Bertand Delannoy, Benoit Gignoux, Olivier Guillaud, Joseph Nloga, Florence Prunier-Bossion, Franck Sibellas

Department of Anaesthesiology and Intensive Care, Hôpital Edouard Herriot, Lyon:

Paul Abraham, Cyril Bidon, Thomas Rimmele

Department of Anaesthesiology and Intensive Care, Centre Hospitalier Lyon Sud, Lyon:

Marie-Hélène Bruge-Ansel, Arnaud Friggeri

Department of Anaesthesiology and Intensive Care, Neurological Hospital - Hospices Cilvils de Lyon, Lyon:

Anne-Claire Lukaszewicz

Department of Anaesthesiology and Intensive Care, Croix Rousse Hospital, Lyon:

Mikhail Dziadzko

Department of Anaesthesiology and Intensive Care, Hospital Nord, Marseille:

Marc Leone, Zoe Meresse, Bruno Pastene

Department of Anaesthesiology and Intensive Care, Hospital Center of Martigues, Martigues:

Isabelle Odin

Clinical Research Department, Clinique Saint Jean, Montpellier:

Aurelien Bonnal, Nicolas Bouic, Pierre Trinh Duc, Thomas Pillant, Fabien Riboulet

Department of Anaesthesiology, GHRMSA, Mulhouse:

Samuel Degoul, Nicolas Saumier, Marion Wasilewski

Department of Anaesthesiology, University Hospital Nantes, Nantes:

Karim Asehnoune, Antoine Roquilly

Department of Anaesthesiology and Intensive Care, University Hospital Pitié-Salpêtrière, Paris:

Pauline Glasman, Louis Puybasset

Department of Anaesthesiology and Intensive Care, Hospital Saint Antoine, Paris:

Fanny Garnier, Franck Verdonk

Department of Anaesthesiology and Intensive Care, Cochin University Hospital, Paris:

Charles Marc Samama, Line Towa

Department of Anaesthesiology and Intensive Care, Saint-Louis Hospital, Paris:

Alice Blet

Department of Anaesthesiology and Intensive Care, University Hospital of Poitiers, Poitiers:

Stéphanie Barrau, Matthieu Boisson, Bertrand Debaene, Denis Frasca, Nadia Imzi

Department of Anaesthesiology and Intensive Care, Private Hospital Claude Galien, Quicy sous Sénart:

Bernard Delvaux, Davy Huynh, Olivier Maupain, Luc Mercadal, Nabil Zanoun

Department of Anaesthesiology and Intensive Care, University Hospital of Reims, Reims:

Armelle de Baene, Catherine Boulay-Maninovsky, Olivier Fernandes, Agathe Giltaire, Philippe Gomis, Jean-Marc Malinovsky, François-Xavier Romain

Department of Anaesthesiology, Santé Atlantique, Saint Herblain:

Astrid Calmelet, Ségolène Dupont, David Gouraud, Sophie Millet, Frédéric Simonneau

Department of Anaesthesiology, Clinique Trenel, Sainte colombe les viennes:

Francoise Charret

Department of Anaesthesiology and Critical Care Medicine, University Hospital Saint-Etienne Nord, Saint-Etienne:

Charlène Couturier, Julien Lanoiselée, Estelle Lornage, Jeremy Mallard, Ryan Milati, Sylvie Passot, Sylvain Vallier

Department of Anaesthesiology, Mutualist Surgical Clinic of Saint Etienne, Saint-Etienne:

Mihaela Luiza Agavriloaia, Quentin Badoux, Mehdi Lewandowski, Yanis Mermet, Denis Michel

Department of Anaesthesiology, GH Selestat-Obernai, Selestat:

Olga Kiskira

Department of Anaesthesiology, Hospital Foch, Suresnes:

Sherifa Adjavon, Virginie Dumans, Morgan le Guen, Julien Josserand, Sabrina Ma

Department of Anaesthesiology, Centre Hospitalier de bigorre, Tarbes:

Jeremy Castanera, Benjamin Massiera, Philippe Petua

Department of Anaesthesiology and Intensive Care, University Hospital of Toulouse, Toulouse:

Fanny Bounes-Vardon, Gaëlle Bosc, Laëtitia Bosch, Edouard Clermond, Fabrice Ferre, François Labaste, Charlotte Martin, Rémi Menut, Vincent Minville, Mohamed Srairi

Department of Anaesthesiology, Gustave Roussy Cancer Center, Villejuif:

Maria Tarasi, Florent Varin

Société Française d'Anaesthésie et de Réanimation (SFAR) Research Network, Paris.

**Georgia**

*National Coordinator:*

Tamar Macharadze, David Tvildiani Medical University, Tbilisi, Georgia and Leibniz Institute for Neurobiology, Department Systems Physiology of Learning, Magdeburg, Germany and Department of Anaesthesiology and Intensive Care, Otto-von-Guericke-Universität, Magdeburg, Germany.

Department of Anaesthesiology and Intensive Care, Health center Medina, Batumi:

Mzia Beridze

Department of Ophthalmology, Oftalmij, Tbilisi:

George Beria, Gabriel Janashvili

Department of Anaesthesiology, Evex Traumatology Hospital, Tbilisi:

Rusudan Bechvaia

Department of Anaesthesiology and Intensive Care, Tbilisi Referral Hospital, Medical Corporation Evex, Tbilisi:

Ketevan Vibliani

Department of Anaesthesiology, New Hospitals, Tbilisi:

Zurab Lekiashvili

Department of Anaesthesiology and Intensive Care, 5th General Hospital Open heart, Tbilisi:

George Grigolia

Department of Anaesthesiology, Academician Nikoloz Kipshidze Central University Clinic, Tbilisi:

Nikoloz Kvachadze

Department of Anaesthesiology and Intensive Care, Medi Club Georgia, Tbilisi:

Nikoloz Kachibaia

Intensive Care Unit, Zugdidi Referral hospital, Medical Corporation Evex, Zugdidi:

Maka Malania

**Germany**

*National Coordinator:*

Mark Coburn, Department of Anaesthesiology, University Hospital RWTH Aachen, Aachen and Department of Anaesthesiology and Intensive Care Medicine, University Hospital Bonn, Bonn

Department of Anaesthesiology, University Hospital RWTH Aachen, Aachen:

Mark Coburn*, Linda Grüßer, Ana Kowark, Rolf Rossaint, Julia Van Waesberghe, Sebastian Ziemann; (*further affiliation: Department of Anaesthesiology and Intensive Care Medicine, University Hospital Bonn, Bonn)

Department of Anaesthesiology, Intensive Care and Pain management, University Hospital Knappschaftskrankenhaus Bochum, Bochum:

Lars Bergmann, Hartmuth Nowak, Günther Oprea, Katharina Rump, Matthias Unterberg

Department of Anaesthesiology and Intensive Care, St. Josef-Hospital Bochum, Bochum:

Heike Vogelsang, Mitja Klutzny

Department of Anaesthesiology and Intensive Care Medicine, University Hospital Bonn, Bonn:

Mark Coburn, Claudia Neumann, Martin Soehle, Maria Wittmann

Department of Anaesthesiology and Intensive Care, University Hospital Carl Gustav Carus Dresden, Dresden:

Martin Scharffenberg, Jakob Wittenstein

Department of Anaesthesiology, Heinrich-Heine University Düsseldorf, Düsseldorf:

Jonas Hinterberg, Peter Kienbaum, Giovanna Lurati-Buse, Frank Nickel, Maximilian Schäfer

Department of Anaesthesiology, Intensive Care Medicine and Pain Therapy, University Hospital Frankfurt, Goethe University, Frankfurt:

Simone Lindau, Patrick Meybohm*, Florian Piekarski; (* further affiliation: Department of Anaesthesiology, University Hospital Wuerzburg, Wuerzburg)

Department of Anaesthesiology and Intensive Care, Hannover Medical School, Hannover:

Hans-Joerg Gillmann, Theresa Anna Kaufhold, Wolfgang Koppert, Andreas Leffler, Hans-Peter Reiffen, Diana Rudolph, Henning Starke, Thomas Stueber

Department of Anaesthesiology, Intensive Care, Pain Management and Palliative Care, Marien Hospital Herne, Herne:

Petra Bischoff, Heinz Haberecht, Heiko Plehn

Clinic for Anaesthesiology and Intensive Care Medicine, Jena University Hospital, Jena:

Michael Bauer, Andreas Kortgen, Christoph Sponholz

Department of Anaesthesiology and Intensive Care, University Hospital Schleswig-Holstein Campus Lübeck, Lübeck:

Uwe Krüger, Sabine Müller-Esch, Mareike Otto, Christian Rempf, Christian Schmidt, Dunja Schumacher

Department of Anaesthesiology and Intensive Care, University Hospital Marburg, Marburg:

Juliane Blazek, Christin Büttner, Andrea Leibeling, Dirk Rüsch, Hinnerk Wulf

Department of Anaesthesiology and Intensive Care, Johannes Wesling Clinic Minden, Minden:

Karsten Burow, Eugen Amir El-Hilali, Christian Greke, Paul Großmann, Mario Kluth, Regina Schulz

Department of Anaesthesiology and Intensive Care, Technical University of Munich, School of Medicine, Klinikum rechts der Isar, Munich:

Sofiane Dridi, Ivana Popovska, Andrés Brenes, Andreas Ranft, Pia Feddersen, Dominik Gerstmeyer, Philippe Fthenakis, Gerhard Schneider, Dirk Miketta

Department of Anaesthesiology, LMU University Hospital, LMU Munich, Munich:

Vera von Dossow, Philipp Groene, Dominik Höchter, Klaus Hofmann-Kiefer, Tobias Kammerer, Malte Kamrath, Thomas Saller, Simon Thomas Schaefer, Roland Tomasi, Tobias Wiedemann, Catharina Zeuzem-Lampert, Bernhard Zwissler

Department of Anaesthesiology and Intensive Care, St. Franziskus Hospital, Münster:

Stephan Braune, Mona Brune, Simone Gurlit, André Hemping-Bovenkerk, Michael Möllmann, Mario Santamaria, Leonie Mareike Schirwitz

Department of Anaesthesiology, Intensive Care and Pain Medicine, University Hospital Münster, Münster:

Melanie Meersch, Alexander Zarbock

Department of Anaesthesiology and Intensive Care, Hospital Oldenburg, Oldenburg:

Ulf Guenther

Department of Anaesthesiology and Intensive Care, University Hospital Tübingen, Tübingen:

Stefanie Decker, Berthold Drexler, Silvia Hipp, Pascal Hofmann, Markus Müller, Judith Roth, Miriam Seiß

Department of Anaesthesiology, Intensive Care and Pain management, Hospital group St. Antonius and St. Josef, Wuppertal:

Christian Adam, Ingo Schwartges

Department of Anaesthesiology, University Hospital Wuerzburg, Wuerzburg:

Peter Kranke

**Greece**

*National Coordinator:*

Konstantinos Katsanoulas, Department of Anaesthesiology, Hippokrateion General Hospital, Thessaloniki

Department of Anaesthesiology, Democritus University of Thrace, Alexandroupolis:

Pelagia Chloropoulou

Department of Anaesthesiology, General Hospital of Athens "G. Gennimatas", Athens:

Antonia Andreeva, Antonia Dimakopoulou, Amalia Douma, Iphigeneia Gregoriadou, Evelina Koutsouli, Konstantina Mendrinou

Department of Anaesthesiology, Evangelismos General Hospital, Athens:

Eirini Mavrommati, Anastasios Stathopoulos

Department of Anaesthesiology, “Attikon” University Hospital, School of Medicine, National and Kapodistrian University of Athens, Athens:

Chrysanthi Batistaki, Paraskevi Matsota

Department of Anaesthesiology, Alexandra Hospital, Athens:

Konstantina Kalopita, Vasiliki Skandalou

Department of Anaesthesiology, Onassis Cardiac Surgery Center, Athens:

Marina Balanika

Department of Anaesthesiology and Intensive Care, University Hospital of Ioannina, Ioannina:

Georgios Papathanakos, Petros Tzimas

Department of Anaesthesiology, General Hospital of Kavala, Kavala:

Evgenia Ketikidou, Anastasia Vachlioti

Department of Anaesthesiology, General Hospital of Komotini, Komotini:

Bioulent Kiamiloglou, Evangelia Nikouli

Department of Anaesthesiology, University Hospital of Larissa, Larissa:

Eleni Arnaoutoglou, Konstantina Kolonia, Eleni Laou, Konstantinos Stamoulis, Epaminondas Vlachakis

Department of Anaesthesiology and Intensive Care, University Hospital of Patras, Patras:

Georgios Karpetas, Ioanna Lianou, Maria Spyraki, Irini Tatani

Department of Anaesthesiology, Tzaneio General Hospital of Piraeus, Piraeus:

Eleni Panagiotou, Evangelia Samara

Department of Anaesthesiology, General Hospital Papanikolaou Thessaloniki, Thessaloniki:

Anna Kolesnikova, Freideriki Sifaki, Eirini Zarzava

Department of Anaesthesiology, Hippokrateion General Hospital of Thessaloniki, Thessaloniki:

Athanasios Bampzelis, Eleni Georgopoulou

Department of Anaesthesiology Theagenio Cancer Hospital, Thessaloniki:

Eleni Christidou

Department of Anaesthesiology and Intensive Care, AHEPA University Hospital, Thessaloniki:

Georgia Tsaousi

Department of Cardiovascular Anaesthesiology, "G. Papanikolaoy" General Hospital of Thessaloniki, Thessaloniki:

Maria Nastou

Department of Anaesthesiology, G. Papanikolaou General Hospital, Medical School, Aristotle University of Thessaloniki, Thessaloniki:

Orestis Ioannidis

Department of Anaesthesiology, General Hospital of Veroia, Veroia:

Eugene Dolzenko, Georgia Geleve

Department of Anaesthesiology, General Hospital of Volos "Achillopouleio", Volos:

Eleni Logotheti, Fotios Yfantidis

**Ireland**

*National Coordinator:*

Peter Lee, Department of Anaesthesiology, Bon Secours Hospital, Cork

Department of Anaesthesiology, Intensive Care and Pain management, Cork University Hospital, Cork:

Senbagam Rajamanickam, Shanmuga Ramaswamy, Timothy Switzer

Department of Anaesthesiology, Tallagh University Hospital, Dublin:

Gurmukh Das Punshi, Karthikeyan Srinivasan

Department of Anaesthesiology, Intensive Care and Pain management, St. Vincents University Hospital, Dublin:

Michael Gilmartin, Osmond Morris

**Israel**

*National Coordinator:*

Idit Matot, Department of Anaesthesiology, Intensive Care and Pain management, Tel Aviv Medical Center, Tel Aviv

Department of Anaesthesiology, Perioperative Medicine and Pain Treatment, Shaare Zedek Medical Center, Jerusalem:

Immanuel Buchman, Yaacov Gozal

Department of Anaesthesiology, Galilee Medical Center, Nahariya:

Amar Merissat, Reut Peled, Dafna Willner

Department of Anaesthesiology, Rabin Medical Center Beilinson Hospital, Petach Tikva:

Hila Amichay Chariski, Leonid A. Eidelman, Michal Y. Livne, Eitan Mangoubi

Department of Anaesthesiology, Sheba Academic Medical Center, Ramat Gan:

Haim Berkenstadt, Dina Orlcin, Dana Yahav-Shafir

Department of Anaesthesiology, Intensive Care and Pain management, Tel Aviv Sourasky Medical Center, Tel Aviv:

Rita Aharonov, Anat Cattan, Lior Felman, Idit Matot, Einat Refaeli-Awin, Yohai Steinberg, Wisam Zabeeda

**Macedonia**

*National Coordinator:*

Andrijan Kartalov, Department of Anaesthesiology and Intensive Care, University Clinic for Traumatology, Orthopedics, Anaesthesia, Reanimation, and Intensive Care Medicine, Skopje

Department of Anaesthesiology and Intensive Care, University Clinic for Traumatology, Orthopedics, Anaesthesia, Reanimation, and Intensive Care Medicine, Skopje:

Biljana Kuzmanovska, Filip Naumovski, Marija Toleska

Department of Anaesthesiology and Intensive Care, Clinical Center Mother Teresa, Skopje:

Atanas Sivevski

**Netherlands**

*National Coordinator:*

Xavier Falières, Department of Anaesthesiology, Albert-Schweitzer Hospital, Dordrecht

Department of Anaesthesiology, Albert Schweitzer Hospital, Dordrecht:

Anouk Andriessen, Minke Kortekaas

Department of Anaesthesiology, Maastricht University Medical Center, Maastricht:

Wolfgang Buhre, Roos Van Gorp, Dianne de Korte-de Boer, Valerie Smit-Fun, Maurice Theunissen

Department of Anaesthesiology, Maasstad Hospital, Rotterdam:

Mirjam Droger, Toine van den Enden, Seppe Koopman

Department of Anaesthesiology and Intensive Care, University Medical Center Utrecht, Utrecht:

Marije Marsman, Eva van Schaik

**Poland**

*National Coordinator:*

Jakub Kenig, Department of General, Oncologic and Geriatric Surgery, Jagiellonian University Medical College, Krakau

Department of General, Oncologic and Geriatric Surgery, Jagiellonian University Medical College, Krakau:

Jakub Kenig

**Portugal**

*National Coordinator:*

Rosário Órfão, Department of Anaesthesiology, Hospital and University Center Coimbra, Coimbra

Department of Anaesthesiology, Hospital and University Center Coimbra, Coimbra:

Marta Azenha, Camile Lanzaro, Rosário Órfão

Department of Anaesthesiology, Centro Hospitalar Universitário Lisboa Norte, Lisbon:

Andreia Borrego, Pedro Branquinho, Sofia Fernandes, Miguel Laires, Denise de Noronha

Department of Anaesthesiology, Hospital Center Tâmega and Sousa, Penafiel:

Inês Ferraz, Ana Pires, Joana Silva

**Romania**

*National Coordinator:*

Mihai Stefan, Department II of Cardiac Anaesthesia and Intensive Care, Emergency Institute for Cardiovascular Diseases "Prof. Dr. C.C. Iliescu", UMF Carol Davila, Bucharest

Department of Anaesthesiology and Intensive Care, Emergency University Central Military Hospital, Bucharest:

Dan Corneci, Oana Oprea, Stefan-Vladimir Zahiu

Department of Anaesthesiology and Intensive Care, Fundeni Clinical Institute, Carol Davila "University of Medicine and Pharmacy Bucharest, Bucharest:

Dana Rodica Tomescu

Intensive Care Unit, Clinical Emergency Hospital of Bucharest, Bucharest:

Ioana Marina Grintescu

Department II of Cardiac Anaesthesia and Intensive Care, Emergency Institute for Cardiovascular Diseases "Prof. Dr. C.C. Iliescu", UMF Carol Davila, Bucharest:

Daniela Filipescu, Mihai Stefan

Department of Anaesthesiology and Intensive Care, County Emergency Hospital Cluj, Cluj:

Elena Stefanaescu

**Russian Federation**

*National Coordinator:*

Victoria Khoronenko, Department of Anaesthesiology and Intensive Care, P.A. Herzen Moscow Cancer Research Institute, Moscow

Department of Anaesthesiology and Intensive Care, Chelyabinsk Regional Clinical Center of Oncology and Nuclear Medicine, Chelyabinsk:

Andrey Vazenin

Department of Anaesthesiology and Intensive Care, P.A. Herzen Moscow Cancer Research Institute, Moscow:

Danil Baskakov, Victoria Khoronenko

Department of Anaesthesiology, NN Blokhin National Medical Cancer Research Center, Moscow:

Dmitry Tipisev

Department of Anaesthesiology and Intensive Care, Federal State Autonomous Institution "N.N. Burdenko National Scientific and Practical Center for neurosurgery" of the Ministry of Healthcare of the Russian Federation, Moscow:

Ksenia Kozlova

**Serbia**

*National Coordinator:*

Miodrag Milenovic, Faculty of Medicine, University of Belgrade, Belgrade, Serbia and Department of Anaesthesiology and Resuscitation, Emergency Center - Clinical Center of Serbia, Belgrade

Department of Anaesthesiology and Intensive Care, CHC Bezaniska kosa, Belgrade:

Olivera Marinkovic, Ana Sekulic

Department of Anaesthesiology, Emergency Center, Clinical Center of Serbia, Belgrade:

Miodrag Milenovic, Marija Rajkovic

Department of digestive surgery, Center for Anaesthesia, Clinical Center of Serbia, Belgrade:

Marija Djukanovic

Department of Anaesthesiology, Clinic for ENT and maxillofacial surgery, Clinical Center of Serbia, Belgrade:

Jovanka Nikolic

Department of Anaesthesiology, Clinic for orthopedics surgery and traumatology, Clinical Center of Serbia, Belgrade:

Svetlana Sreckovic

Department of Anaesthesiology, Clinic for plastic surgery, Clinical Center of Serbia, Belgrade and Medical Faculty, University of Belgrade, Belgrade:

Marina Stojanovic

Department of Anaesthesiology, Urology Hospital of Clinical Center Serbia, Belgrade:

Nebojsa Ladjevic, Jelena Jovicic

Department of Anaesthesiology and Intensive Care, Cardiovascular Institute Dedinje Belgrade:

Dragana Unic-Stojanovic

Department of General Surgery, Clinical Center Nis, Nis:

Biljana Stosic

Department of Anaesthesiology and Intensive Care, General Hospital Sremska Mitrovica, Sremska Mitrovica:

Aleksandra Bulasevic

**Spain**

*National Coordinator:*

Marina Soro, Department of Anaesthesiology and Intensive Care, Hospital Clinico Universitario, Valencia

Department of Anaesthesiology and Intensive Care, University Hospital Fundación Alcorcón, Alcorcón:

Alma M. Espinosa-Moreno, Jose I. García-Sánchez, Beatriz Martín-Vaquerizo, Clara Morandeira-Rivas, Diana Zamudio

Department of Anaesthesiology, Hospital de la Santa Creu i Sant Pau, Barcelona:

Victoria Baños, Mireia Rodriguez, Selene Martinez, Nerea Guadalupe, Gracia Herranz, Javier Baute, Vanaesa Madrona, Roser de Jose, Jordi Miralles, Alfred Merten, Rolando Muñoz, Anabel Delgado, Victoria Moral

Department of Anaesthesiology, Consorci Sanitari Integral-General de l’Hospitalet, Barcelona:

Aleix Carmona Blesa, Sara Espejo, Laura Grau Torredeflot, Alejandro Romero Fernández, Maria Sanabra, Pere Serra Pujol

Department of Anaesthesiology, University Hospital Sagrat Cor, Barcelona:

Maria Jose Alvira Uribe, Astrid Alvarez Perez, Espedito Brunetto, Federica Castelli, Jorge Gonzalez Aguirre, Adriana Herivas Villar, Guido Munoz Rojas

Department of Anaesthesiology, Consorci Sanitari Integral- Hospital de Sant Joan Despí Moises Broggi, Barcelona:

Aleix Carmona Blesa, Laura Grau Torredeflot, Natalia Montero, Alejandro Romero Fernández, Maria Sanabra

Department of Anaesthesiology, Consorci Sanitari Integral-Hospital Dos de Maig, Barcelona:

Laura Grau Torredeflot, Alejandro Romero Fernández, Maria Sanabra, Pere Serra Pujol

Department of Anaesthesiology and Intensive Care, General University Hospital Ciudad Real, Ciudad Real:

Víctor Baladrón González

Department of Anaesthesiology and Intensive Care, University Hospital of Gran Canaria Doctor Negrin, Las Palmas de Gran Canaria:

Ángel Becerra-Bolaños, Aurelio Rodríguez-Pérez, Luis Santana-Ortega, Vanaessa Suárez-Romero, María Luisa Torres-Machí

Department of Anaesthesiology and Intensive Care, Hospital of Leon, Leon:

Javier Ferrero de Paz, Jose Miguel Marcos -Vidal, Ana Martín Garcia, María Merino García, Consuelo Rego Diaz

Department of Anaesthesiology, Hospital San Pedro, Logrono:

Ana Crespo Santiago, Lourdes Ferreira Laso, Felix Lobato Solores

Department of Anaesthesiology and Intensive Care, Hospital Gregorio Marañon, Madrid:

Alba Burgos, Alberto Calvo, Patricia Cruz, Carmen Fernández, Ignacio Fernández, Ignacio Garutti, Fernando Higuero, David Martinez, Patricia Piñeiro

Department of Anaesthesiology, Intensive Care and Pain management, University Hospital La Princesa, Madrid:

Sonia Expósito Carazo, Rosa Méndez Hernández, Mar Orts Rodríguez, Fernando Ramasco Rueda

Department of Anaesthesiology and Intensive Care, Infanta Leonor University Hospital, Madrid:

Ane Abad-Motos, Javier Ripollés-Melchor

Department of Anaesthesiology, Hospital Ramón y Cajal, Madrid:

Carmen Pastor López

Department of Anaesthesiology and Intensive Care, University La Fe Hospital Valencia, Valencia:

Pedro Charco, Sara Perez-Palao, Laura Sancho-Iñigo, Nasara Segura, Marina Soro, Esther Utrera

Department of Anaesthesiology and Intensive Care, University Hospital of Araba, Vitoria-Gasteiz:

Ania Albinarrate, Ana María Fondarella

Department of Anaesthesiology and Intensive Care, University Hospital "Miguel Servet", and Aragon Institute for Health Research (IIS Aragon), Zaragoza:

Lucia Gallego-Ligorit

Department of Anaesthesiology and Intensive Care, University Hospital "Miguel Servet", Zaragoza:

Luisa Lacosta Torrijos

**Switzerland**

*National Coordinator:*

Nicolai Goettel, Department of Anaesthesia, Prehospital Emergency Medicine and Pain Therapy, University Hospital Basel, Basel, Switzerland and Department of Clinical Research, University of Basel, Basel, Switzerland

Department of Anaesthesia, Prehospital Emergency Medicine and Pain Therapy, University Hospital Basel, Basel:

Oliver Bandschapp, Andrea A. Blum, Nicolai Goettel, Esther Seeberger, Luzius A. Steiner, Alessandra E. Thomann

Department of Anaesthesiology, Kantonsspital Baselland Liestal, Liestal:

Seraina Frei, Susan Hoehn

Department of Anaesthesiology and Intensive Care, St. Anna Clinic, Lucerne:

Bertram Baenziger, Giuliana Capaldo, Daniel Christ, Ramon Doerig, Daniel Hodel

Department of Anaesthesiology and Intensive Care, Hospital Limmattal, Schlieren:

Andreas Weiss, Lukas Witt

Department of Anaesthesiology, Hospital Solothurn, Solothurn:

Philippe Schumacher, Dirk André Siebing

**Turkey**

*National Coordinators:*

Zerrin Sungur, Department of Anaesthesiology, Istanbul University, Istanbul Faculty of Medicine, Istanbul

Zekeriyya Alanoglu, Department of Anaesthesiology and Intensive Care, Ankara University Medical School, Ankara

Department of Anaesthesiology and Intensive Care, Ufuk University Dr. Ridvan Ege Hospital, Faculty of Medicine, Ankara:

Seyma Orcan Akbuz, Zuleyha Kazak Bengisun, Baturay Kansu Kazbek, Ulku Ceren Koksoy, Engin Zafer Terzi, Hakan Yilmaz,

Department of Anaesthesiology and Intensive Care, Ankara University Faculty of Medicine, Ibni Sina Hospital, Ankara:

Neslihan Alkis, Sanem Cakar Turhan, Basak Ceyda Meco, Konul Hajiyeva, Cigdem Yildirim Guclu

Department of Anaesthesiology and Intensive Care, University of Health Sciences Diskapi Yildirim Beyazit Education and Training Hospital, Ankara:

Jülide Ergil, Emine Unal Ceran

Department of Anaesthesiology and Intensive Care, Ankara University Cebeci Hospital, Ankara:

Menekse Ozcelik

Department of Anaesthesiology and Intensive Care, Balikesir Atatürk City Hospital, Balikesir:

Atik Bülent, Kilinc Gökhan

Department of Anaesthesiology and Intensive Care, Kocaeli Derince Training and Research Hospital, Derince/Kocaeli:

Kemal Tolga Saracoglu (now affiliated to Clinic of Anaesthesiology and Intensive Care Health Sciences University Kartal Dr. Lutfi Kirdar Training and Research Hospital, Istanbul)

Department of Anaesthesiology and Intensive Care, TR Ministry of Health Erzurum Palandoken State Hospital, Erzurum:

Bunyamin Kir

Department of Anaesthesiology, Istanbul University, Istanbul Faculty of Medicine, Istanbul:

Kemalettin Koltka, Nükhet Sivrikoz

Department of Anaesthesiology, Marmara University Pendik Training and Research Hospital, Istanbul:

Pelin Corman Dincer

Department of Anaesthesiology, Baltalimani Metin Sabanci Bone and Joint Diseases Education and Research Hospital, Istanbul:

Nur Canbolat

Department of Anaesthesiology and Intensive Care, Dr. Siyami Ersek Training and Research Hospital, Istanbul:

Turkan Kudsioglu

Department of Anaesthesiology, Sağlık Bilimleri University Tepecik Education and Research Hospital, Izmir:

Gaye Aydin, Ceren Aygün Mucuoglu

Department of Anaesthesiology, Dokuz Eylül University Faculty of Medicine, Izmir:

Duriye Gul Inal, Semih Kucukguclu

Department of Anaesthesiology and Intensive Care, Konya Education and Research Hospital, Konya:

Ayse Ilksen Egilmez, Betul Kozanhan, Munise Yildiz

Department of Anaesthesiology, Baskent University Konya Research Center, Konya:

Hüseyin Ulas Pinar

Department of Anaesthesiology and Reanimation, Recep Tayyip Erdogan University School of Medicine, Rize:

Başar Erdivanlı, Ayşe Hızal, Emre Karagöz, Hızır Kazdal, Abdullah Özdemir

Department of Anaesthesiology and Intensive Care, Sakarya University, Sakarya:

Ayca Tas Tuna

Department of Anaesthesiology, Republic of Turkey Ministry of Health Tunceli State Hospital, Tunceli:

Gamze Gulgun

**Ukraine**

*National Coordinator and Investigator:*

Dolya Oleg, Department of Anaesthesiology and Intensive Care, Zaporozhye State Medical University, Zaporozhye
