# Supplementary material for: Pre-Interventional Risk Assessment in The Elderly (PIRATE): Development of a scoring system to predict 30-day mortality using data of the Peri-Interventional Outcome Study in the Elderly
Source: PLoS One. 2023 Dec 21;18(12):e0294431. doi: 10.1371/journal.pone.0294431 (PMC10734910; doi:10.1371/journal.pone.0294431)
Supplement: S1 Table — (DOCX) [file pone.0294431.s002.docx]

**S2 Table. Example application of the PIRATE tool.**

As a first example, consider a patient admitted to hospital from home (living independently). The patient requires a major surgery that was initially planned.

A second example considers a patient living independently. The patient was admitted to hospital to undergo a non-planned minor surgery.

The third and fourth examples consider patients living (medically) assisted. The patients were admitted to hospital to undergo major surgeries that were either elective (patient 3) or non-elective (patient 4).

| **Risk Factor** | **Severity** | **Urgency** | **Living Conditions** | **Total Score** | **Estimated 30-day death probability** |
| --- | --- | --- | --- | --- | --- |
| **Patient 1** | *Major*  *+1* | *Elective*  *+0* | *independent*  *+0* | *1* | *2.33%* |
| **Patient 2** | *Minor*  *+0* | *Non Elective*  *+2* | *independent*  *+0* | *2* | *4.20%* |
| **Patient 3** | *Major*  *+1* | *Elective*  *+0* | *(medically) assisted*  *+2* | *3* | *7.51%* |
| **Patient 4** | *Major*  *+1* | *Non Elective*  *+2* | *(medically) assisted*  *+2* | *5* | *22.78%* |
